# Modulation of the Tumor Microenvironment by CXCR4 Antagonist-Armed Viral Oncotherapy Enhances the Antitumor Efficacy of Dendritic Cell Vaccines against Neuroblastoma in Syngeneic Mice

**DOI:** 10.3390/v10090455

**Published:** 2018-08-26

**Authors:** Marcin Komorowski, Joanna Tisonczyk, Agnieszka Kolakowska, Ryszard Drozdz, Danuta Kozbor

**Affiliations:** 1 Department of Immunology, Roswell Park Comprehensive Cancer Center, Elm and Carlton Streets, Buffalo, NY 14263, USA; joanna.tisonczyk@uj.edu.pl (J.T.); akolakowska@pzh.gov.pl (A.K.); Danuta.Kozbor@roswellpark.org (D.K.); 2 Department of Medical Diagnostics, Faculty of Pharmacy, Jagiellonian University Medical School, Medyczna 9, 30-688 Cracow, Poland; ryszard.drozdz@uj.edu.pl; 3 Department of Virology, National Institute of Public Health-National Institute of Hygiene, Chocimska 24, 00-791 Warsaw, Poland

**Keywords:** oncolytic viruses, dendritic cell vaccines, tumor microenvironment, neuroblastoma

## Abstract

The induction of antitumor immune responses in tumor-bearing hosts depends on efficient uptake and processing of native or modified tumors/self-antigens by dendritic cells (DCs) to activate immune effector cells, as well as the extent of the immunosuppressive network in the tumor microenvironment (TME). Because the C-X-C motif chemokine receptor 4 (CXCR4) for the C-X-C motif chemokine 12 (CXCL12) is involved in signaling interactions between tumor cells and their TME, we used oncolytic virotherapy with a CXCR4 antagonist to investigate whether targeting of the CXCL12/CXCR4 signaling axis in murine neuroblastoma cells (NXS2)-bearing syngeneic mice affects the efficacy of bone marrow (BM)-derived DCs loaded with autologous tumor cells treated with doxorubicin for induction of immunogenic cell death. Here, we show that CXCR4 antagonist expression from an oncolytic vaccinia virus delivered intravenously to mice with neuroblastoma tumors augmented efficacy of the DC vaccines compared to treatments mediated by a soluble CXCR4 antagonist or oncolysis alone. This study is the first demonstration that modulating the tumor microenvironment by an armed oncolytic virus could have a significant impact on the efficacy of DC vaccines, leading to the generation of effective protection against neuroblastoma challenge.

## 1. Introduction

Neuroblastoma (NB) is a usually poorly differentiated neoplasm which originates from neural crest cells and has high potential for metastasis. It is one of the most common malignancies among infants and young children. The uniqueness of NB lies in its significant heterogeneity. In some patients it can undergo spontaneous regression despite the high expansion of the disease, whereas it can show a very aggressive behavior resulting in a poor long-term survival rate in others [1,2]. One of the prognosis factors for NB is the infiltration of tumor cells to bone marrow (BM). This usually indicates an advanced stage of the disease and is associated with a poor prognosis [3,4]. The C-X-C motif chemokine receptor 4 (CXCR4) expression has been shown in tumors associated with BM metastases such as breast cancer [5], prostate cancer [6], neuroblastoma [7,8], and rhabdomyosarcoma [9]. It has been reported that neuroblastoma cell lines express CXCR4 and bind the CXCR4 ligand stromal-derived factor-1 (SDF-1), also known as C-X-C motif chemokine 12 (CXCL12), which induces tumor cell migration. Moreover, CXCR4 expression is higher in NB tumor cells from patients with BM metastases compared to those with localized tumors [3,8,10]. All these studies support the hypothesis that specific CXCL12 chemokines/CXCR4 receptor signaling may play important roles in NB tumor cell invasion and metastasis. In addition, a high level of CXC12 in the tumor may also impact the ability of immunotherapeutic approaches to induce antitumor immune responses because CXCL12 has been shown to augment the immunosuppressive network in the tumor microenvironment (TME), which impedes immune mechanisms of tumor destruction [11,12]. Therefore, modulation of the CXCL12/CXCR4 axis in NB tumors could impact multiple aspects of tumor pathogenesis, including immune dysregulation. Because the immunosuppressive network in the TME may modulate progression of tumor growth and may also affect immunotherapeutic strategies including tumor-specific dendritic cell (DC) vaccines, we investigated whether targeted delivery of a CXCR4 antagonist by oncolytic virotherapy augments DC vaccine efficacy.

Oncolytic viruses, including the oncolytic vaccinia virus (OVV), are emerging as potent therapeutics, targeting tumor cells for destruction and simultaneously promoting inflammation in the TME and anti-tumor immunity [13,14,15,16,17]. With the recent FDA approval of talimogene laherparepvec (T-VEC; IMLYGIC) for melanoma patients [18] and additional viruses in both pre-clinical and clinical development [19], there is a need to establish the optimal approaches for oncolytic virotherapy to enhance anti-tumor immunity. Previously, we designed a tumor cell-targeted therapy that delivered a CXCR4 antagonist expressed in the context of the murine Fc fragment of IgG2a via an OVV (OVV-CXCR4-A-Fc) [13,14]. The OVV replicates in cancer cells, selectively causing activation of cellular EGFR/Ras signaling, thymidine kinase elevation, and type-1 interferon (IFN) resistance [20]. The OVV turned out to be an excellent vector for CXCR4 antagonist delivery. It has large transgene-encoding capacity, evolved mechanisms for intravenous stability, and can spread to distant tissues. Moreover, it has a strong lytic ability and has proven safety in humans as a vaccine [21,22,23,24]. Furthermore, as a result of viral infection, inflammatory responses are triggered in order to clear the virus. Consequently, important cellular and viral danger signals are released, resulting in reduction of tumor-mediated immune suppression and mediating tumor destruction [16,25,26]. Our previous results, which demonstrated that OVV-CXCR4-A-Fc treatment of mice with orthotropic breast and ovarian tumors exhibits increased efficacy over oncolysis alone [15,16,27], prompted us to examine the effect of the armed oncolytic virotherapy on induction of protective immune responses with DC vaccines presenting tumor-associated antigens from murine neuroblastoma cells (NXS2).

For immunization, we have used DCs pulsed with whole tumor lysates prepared from NXS2 tumor cells treated with doxorubicin (Dox) because anthracycline-treated tumor cells are effective in eliciting anticancer immune responses due to the induction of immunogenic cell death (ICD) associated with the spatiotemporal release of danger-associated molecular patterns (DAMPs) from dying cells [28]. The critical DAMPs are endogenous factors that translocate to abnormal cellular compartments in a temporal cascade [29,30], and after translocation they interact with receptors and stimulate cellular and cytokine responses against cancer-derived antigens. For example, upon Dox-induced endoplasmic reticulum (ER) stress, Ca^2+^ efflux, and accumulation of reactive oxygen species, calreticulin (CRT) complexes with a lumenal protein of the ER (ERp57) during transport from the ER and docks onto CD91/low-density lipoprotein receptor related protein 1 (LRP1) [31]. Ecto-CRT/ERp57 signals through CD91 and scavenger receptor class A (SR-A) as well as scavenger receptor expressed by endothelial cell-I (SREC-I) [32,33] on innate immune cells, promoting phagocytosis and nuclear factor kappa-light-chain-enhancer of activated B cells (NF-kB) pathway activation [34].

Here, we showed that the DC vaccines loaded with Dox-treated NXS2 tumor cells were highly efficient in inducing antitumor immune responses in vaccinated mice both in prophylactic and therapeutic settings. The antitumor efficacy of the therapeutic vaccines was largely dependent on the reduction of tumor load and reprogramming of the TME by OVV-CXCR4-A-Fc, suggesting that the armed OVV can augment the vaccine-induced immune responses.

## 2. Materials and Methods 

### 2.1. Animals and Cell Lines

All animal studies were approved by the Institutional Animal Care and Use Committee of the Roswell Park Comprehensive Cancer Center (RPCCC, Buffalo, NY, USA) on 22 November 2013, and the project identification code was 1018M. Adult (6–8 weeks of age) female A/J mice were purchased from The Jackson Laboratory (Sacramento, CA, USA). Adult (6–8 weeks of age) female C.B-*Igh-1^b^*/IcrTac-*Prkdc^scid^* mice were purchased from the Laboratory of Animal Resources at the RPCCC. The murine NXS2 neuroblastoma cell line was provided by the Dr. R. A. Reisfeld, Scripps Research Institute, La Jolla, CA, USA. Cells were cultured with RPMI 1640 medium (Thermo Fisher Scientific, Grand Island, NY, USA) enriched with 10% fetal calf serum (FCS; Invitrogen, Carlsbad, CA, USA). Cells were cultured in monolayer at 37 °C and 5% CO_2_. The cell line was shown to be major histocompatibility complex (MHC) class I syngeneic to A/J mice by its H-2K^k^-positive/H-2K^b^-negative phenotype [35,36].

### 2.2. Viruses

The Western Reserve strain oncolytic vaccinia virus with disrupted thymidine kinase (*TK*) and vaccinia growth factor (*VGF*) genes was used in this study. The virally expressed CXCR4 antagonist consists of eight amino acids corresponding to the N-terminal sequence of CXCL12 with modified P to G (KGVSLSYR). The generation and characterization of vaccinia viruses expressing the CXCR4 antagonist in the context of murine Fc fragment of IgG2a have been previously described [16,37].

### 2.3. Generation of Bone Marrow (BM)-Derived DC and In Vitro phagocytosis Assays

RPMI 1640 medium enriched with 10% heat-inactivated fetal calf serum (FCS; Invitrogen), sodium pyruvate, 50 μmol/L 2-mercaptoethanol (Sigma-Aldrich, Saint Louis, MO, USA), 10 mmol/L *N*-2-hydroxyethylpiperazine-*N*9-2-ethanesufonic acid (HEPES, pH 7.4), and penicillin/streptomycin (Invitrogen) was used to flush bone marrow (BM) cells from the tibias and femurs of A/J mice. After one centrifugation, the cell pellet was resuspended in Tris-ammonium chloride for 2 min to lyse red blood cells, and centrifuged once more. In the next step, BM cells (1 × 10^6^ cells/mL) were cultured in medium supplemented with 10 ng/mL GM-CSF at 37 °C and 5% CO_2_ for 7 days. The medium was replenished every 2–3 days. At the end of the incubation, the non-adherent and loosely adherent cells were harvested, washed, and cocultured for 12 h with 0.5 × 10^6^ NXS2 tumor cells labeled with The CellTracker™ Blue CMF_2_HC fluorescent dye (Thermo Fisher Scientific) at a 1:1 ratio. Then, cells were harvested with versene, washed, and stained with antibody conjugated with allophycocyanin (APC) against mouse CD11c (CD11c-APC). Fluorescence-activated cell sorting (FACS) analysis was performed to assess the phagocytosis. Double-positive cells were considered to be the BM-derived DCs that had phagocytized NXS2 tumor cell debris.

For the DC vaccines preparation, tumor cells were subjected to six freeze–thaw cycles or to 24 h treatment with Dox (10 µM) before coculturing with DCs.

### 2.4. Vaccination of Mice and NXS2 Tumor Challenge

Tumor lysates of Dox-treated NXS2 cells were cocultured with mouse DCs for 12 h, incubated with LPS (1.0 µg/mL) for 1 h to induce maturation through binding to the toll-like receptor 4 (TLR4) [36], washed, and injected intradermally (2 × 10^6^) into A/J mice. For a prophylactic vaccine, female A/J mice (*n* = 5/group) received subcutaneous (SC) injections in the lateral flank with 2 × 10^6^ murine NSX2 neuroblastoma cells on day 8 after DC vaccines. In a therapeutic setting, A/J mice (*n* = 5/group) received SC injections with 2 × 10^6^ NXS2 cells in the lateral flank and 7 days later DC vaccines prepared as mentioned above were injected intradermally into the opposite site. In both experiments unvaccinated A/J mice or A/J mice immunized with DCs loaded with lysates prepared from untreated tumor cells served as controls. An analogical experiment was performed in SCID mice to confirm effectiveness of DCs loaded with Dox-treated NXS2 lysates as a prophylactic vaccine. Tumor growth was monitored by measuring SC tumors once to thrice a week with a microcaliper and determining tumor volume (width × length × width/2 = mm^3^). Survival was defined as the point at which mice were sacrificed due to extensive tumor growth.

### 2.5. Treatment of Established Tumors

A/J mice (*n* = 5/group) received SC injections with 2 × 10^6^ NXS2 cells and treated with OVV-CXCR4-A-Fc or OVV-Fc (10^8^ PFU delivered intravenously, IV) once the tumor volumes reached ~100 mm^3^. Control mice received PBS or UV-inactivated virus. For therapeutic vaccine studies, 7 days after oncolytic virotherapy treatment the NXS2 tumor-bearing mice were injected intradermally with DCs loaded with Dox-treated NXS2 lysates (2 × 10^6^). Tumor growth was monitored by measuring SC tumors once to thrice a week with a microcaliper. 

### 2.6. Flow Cytometry

For flow cytometry experiments, an LRS II flow cytometer (BD Biosciences, San Jose, CA, USA) was used. To establish the percentage of apoptotic/necrotic NSX2 tumor cells after incubation with 10 µM Dox, Annexin V conjugated with fluorescein isothiocyanate (Annexin V-FITC) and LIVE/DEAD fixable violet (Thermo Fisher Scientific) staining was performed. Tumor cells were analyzed for cell surface expression of ecto-CRT by staining with rabbit anti-mouse CRT monoclonal antibody (anti-mouse CRT mAb, Abcam, Cambridge, MA, USA) followed by staining with APC-conjugated goat anti-rabbit secondary antibody (Santa Cruz Biotechnology, Santa Cruz, CA, USA). The analysis of the TME was performed on single-cell suspensions prepared from tumors harvested 8 days after completion of the treatments. Before specific antibody staining, cells were incubated with Fc blocker (anti-CD16/CD32 mAb) for 10 min, followed by LIVE/DEAD Fixable Violet Dead Cell stain kit (Thermo Fisher Scientific) to assess live/dead cells. For a phenotypic analysis of tumor-infiltrating leukocytes, the following specific antibodies were used: rat mAbs against mouse CD11b-APC, Ly6G conjugated with phycoerythrin (Ly6G-PE), Ly6C-FITC, CD45-APC-Cy7, CD4-PECy5, CD25-FITC, CD8-PECy5, IFN-γ-PE, CD11c-APC, CD86-FITC (BD Pharmingen, San Jose, CA, USA), Foxp3-AlexaFluor 647 (Thermo Fisher Scientific), and F4/80-FITC (BioLegend, San Diego, CA, USA). To determine the percentages of CD8^+^ T cells expressing IFN-γ or CD4^+^ T cells expressing Foxp3, intracellular staining using BD Pharmingen Transcription Factor Buffer Set (BD Biosciences) was used. Intracellular staining with PE-conjugated rat mAb against mouse interleukin 10 (IL-10-PE, BD Pharmingen) and anti-h/m IL-12/ILp35-PE Ab (R&D Systems, Minneapolis, MN, USA) was performed to assess the percentages of CD11b/F4/80^+^ tumor-associated macrophages (TAM) expressing IL-12 (TAM1) or IL-10 (TAM2). The higher IL-12/IL-10 ratios in TAMs have been associated with reduction of intratumoral recruitment of immunosuppressive elements in favor of immunostimulatory ones [38]. For each staining, the isotype control antibodies were included. WinList 3D 7.1 (Verity Software House, Topsham, ME, USA) was used to perform data analysis.

### 2.7. Statistical Analysis

For statistical analyses GraphPad Prism 6 statistical package (GraphPad Software, La Jolla, CA, USA) was used. The statistical significance of the differences between groups was performed using a two-tailed Student’s *t*-test assuming equal variance. The *P* values for the pairwise group comparisons for average tumor growth were computed using the nonparametric Wilcoxon rank-sum test. Data were presented as mean ± SD, unless otherwise noted. Kaplan–Meier survival plots were prepared, and median survival times were determined for NXS2-challenged groups of mice. Statistical differences in the survival across groups were assessed using the log rank Mantel–Cox method assuming that the order of data sets correspond to equally spaced ordered categories. *p* < 0.05 was considered statistically significant.

## 3. Results

### 3.1. Dox-Induced Apoptosis of NXS2 Tumor Cells is Associated with CRT Exposure and Phagocytosis by DCs

**Figure 1:**
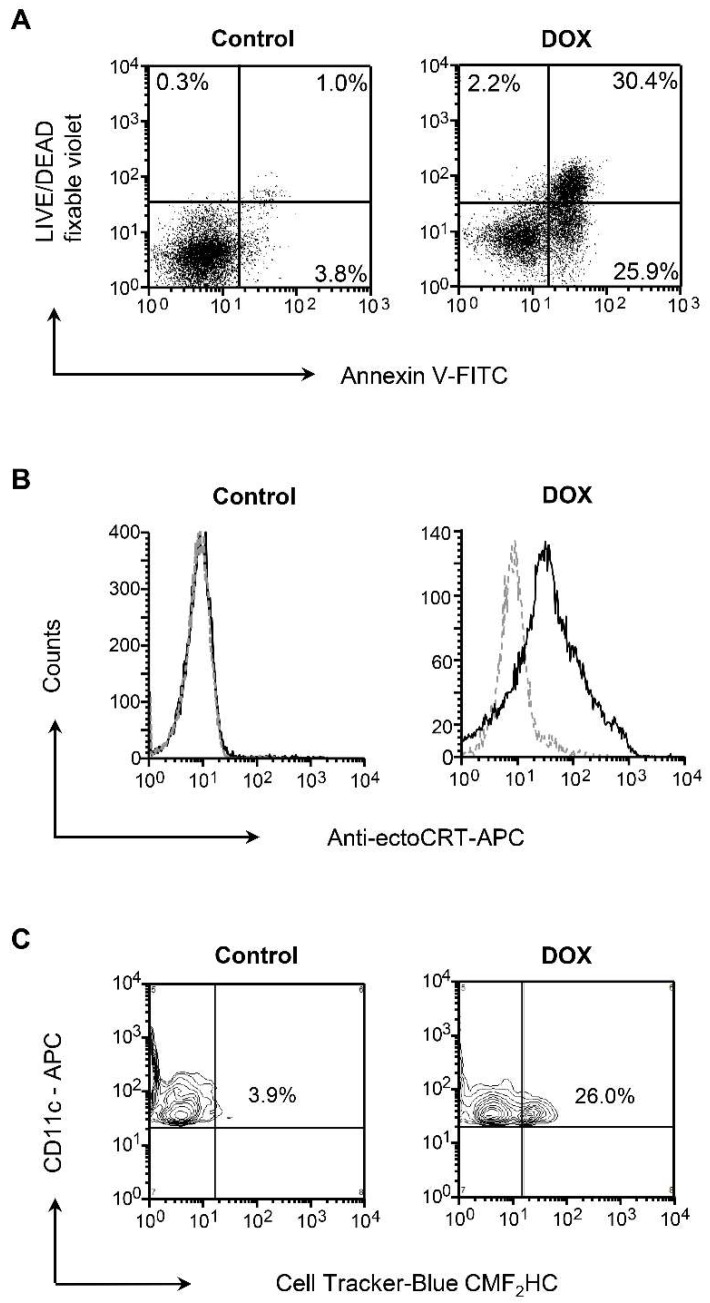
Doxorubicin (Dox)-induced apoptosis is associated with surface exposure of calreticulin (CRT) and phagocytosis of tumor cell debris by bone marrow (BM)-derived dendritic cells (DCs). (**A**) Cell death in murine neuroblastoma tumor cells (NXS2) treated with Dox (10 µM) was determined by staining with Annexin V conjugated with fluorescein isothiocyanate (Annexin V-FITC) and LIVE/DEAD fixable violet to measure the induction of early apoptosis (Annexin V^+^/LIVE/DEAD fixable violet^-^) and late apoptosis/necrosis (Annexin V^+^/LIVE/DEAD fixable violet^+^) by flow cytometry 24 h later. One representative experiment of three independent experiments performed is shown. (**B**) Surface exposure of CRT in NXS2 culture untreated or treated with Dox was determined by flow cytometry after staining with an anti-CRT Ab or an isotype control 24 h after treatment. One representative experiment of three independent experiments performed is shown. (**C**) Mouse BM-derived DCs were co-cultured with cell tracker-blue CMF_2_HC-labeled NXS2 cells (1:1 ratio) for 12 h and analyzed for phagocytosis by flow cytometry. The percentages of CD11c-expressing DCs taking up tumor cells are indicated. One representative experiment of three independent experiments performed is shown.

As the capacity of apoptotic tumor cells to trigger the immune response was found to depend on surface exposure of CRT that confers immunogenicity to otherwise nonimmunogenic cell death, allowing for an optimal anticancer chemotherapy [31], we analyzed the effect of Dox-induced cell death on cell surface expression of CRT in NXS2 neuroblastoma tumors. The induction of apoptosis/necrosis analyzed by flow cytometry with Annexin V-FITC and LIVE/DEAD fixable violet revealed over 50% apoptotic cells after a 24-h treatment with 10 μM of Dox with fewer than 5% necrotic cells (Figure 1A). Consistent with the previous findings that anthracyclines induce a rapid translocation of CRT to the cell surface [31], the Dox-treated cultures were positive for surface exposure of ecto-CRT measured by immunostaining with CRT-specific antibody and flow cytometry (Figure 1B). In view of the established role of surface CRT as an “eat me” signal [39,40], we next investigated the phagocytosis of the treated tumor cells by BM-derived DCs, a condition that is required for mounting immune response against dying tumor cells [41]. For this experiment, mouse BM-derived DCs were cocultured with cell tracker-blue CMF_2_HC-labeled tumor cells (1:1 ratio) for 12 h and analyzed for phagocytosis by flow cytometry after gating for CD11c^+^ DCs. As shown in Figure 1C, NXS2 tumor cells that received the treatment with Dox were over six-fold more efficiently phagocytosed by DCs compared with untreated culture.

### 3.2. Vaccination with DC Vaccines Loaded with Dox-Treated NXS2 Tumor Cells Protects Against Syngeneic Tumor Challenge

**Figure 2:**
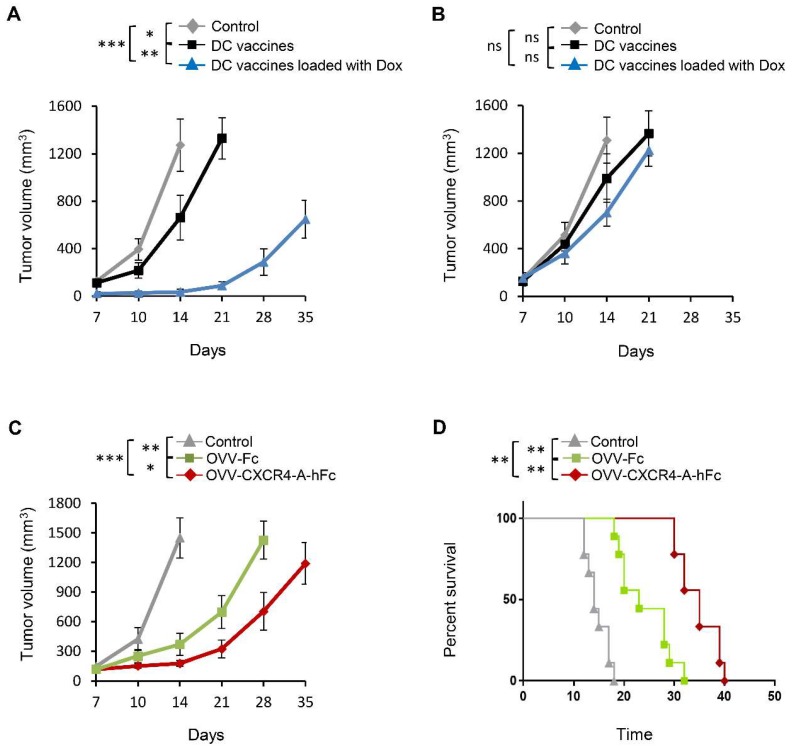
Effect of DC vaccines and oncolytic vaccinia virus (OVV) treatment on tumor growth in A/J mice. (**A**) The immunogenicity of the tumor lysate-pulsed DCs as vaccines in a prophylactic setting. The BM-derived DCs were loaded with Dox-treated NXS2 tumor cell lysates or untreated NXS2 tumor cells and injected intradermally (2 × 10^6^ DCs/dose) into A/J mice. Next, each mouse was injected subcutaneously (SC) in the lateral flank with 2 × 10^6^ NXS2 cells on day 8 after vaccination. Control mice were unvaccinated. Tumor growth was monitored by measuring SC tumor growth with a microcaliper until control mice were euthanized due to extensive tumor burden. Results are presented as mean ± SD of five independent experiments. * *p* < 0.05, ** *p* < 0.01, *** *p* < 0.001. (**B**) The immunogenicity of the tumor lysate-pulsed DCs as vaccines in a therapeutic setting. A/J mice received SC injection with 2 × 10^6^ NXS2 cells in the lateral flank and DCs loaded with Dox-treated NXS2 tumor cell lysates/untreated NXS2 cells were injected intradermally (2 × 10^6^ DCs/dose) into the opposite site 7 days later. Control mice were unvaccinated. Tumor growth was monitored by measuring SC tumor growth with a microcaliper until control mice were euthanized due to extensive tumor burden. Results are presented as mean ± SD of five independent experiments. ns – statistically non-significant. (**C**) The inhibition of tumor growth by the armed OVV. A/J mice (*n* = 6–10/group) received SC injection with 2 × 10^6^ NXS2 cells in the lateral flank. Oncolytic virotherapy with OVV-Fc and OVV-CXCR4-A-hFc delivered intravenously (10^8^ PFU/mouse) was initiated when the subcutaneous tumor volume was ~100 mm^3^. OVV-Fc was used as a specificity control. Control mice were treated with PBS. Tumor progression was monitored by measuring SC tumor growth with a microcaliper until control mice were euthanized due to extensive tumor burden. Results are presented as mean ± SD of five independent experiments. * *p* < 0.05, ** *p* < 0.01, *** *p* < 0.001. (**D**) Survival was defined as the point at which mice were killed because of extensive tumor burden. Kaplan–Meier survival plots were prepared, and significance was determined using the log-rank method. ** *p* < 0.01.

To test the immunogenicity of the whole tumor lysate-pulsed DCs as vaccines in prophylactic and therapeutic settings, BM-derived DCs were loaded with Dox-treated NXS2 or NXS2 lysates prepared from untreated tumor cells and injected intradermally (2 × 10^6^ DCs/dose) into A/J mice. For the prophylactic vaccines (Figure S1), each mouse received an SC injection in the lateral flank with 2 × 10^6^ NXS2 cells on day 8 after vaccination. Unvaccinated animals served as a control group. Protection against tumor growth was interpreted as a sign of successful vaccination and induction of antitumor immunity (Figure 2A), since such protection was not observed in SCID mice (Figure S2). The significant inhibition of tumor growth in mice immunized with Dox-treated cell lysate-loaded DC vaccines compared to those treated with the untreated tumor cells (*p* < 0.001) suggests that the Dox treatment of tumor cells led to upregulation of factors associated with ICD that could potentiate the benefits of direct tumor cell killing by augmenting the induction of antitumor immunity. However, the same experiments performed in NXS2 tumor-challenged mice (therapeutic setting, Figure S3) demonstrated that the vaccine formulation was not effective in inhibiting tumor growth when delivered 7 days after tumor challenge (Figure 2B), which could be due to the fast rate of tumor growth associated with the immunosuppressive network in the TME.

### 3.3. OVV-CXCR4-A-Fc Treatment Inhibits Growth of NXS2 Tumors Associated with Decreases in the Immunosuppressive Networks in the TME

The disparity between the antitumor efficacies between these two treatments suggested that the tumor load together with the immunosuppressive network in the TME could abolish vaccine efficacy. This, together with the accumulating evidence that the chemokine CXCL12 pathway increases tumor resistance to both conventional therapies and biological agents [11,15,16,42] prompted us to explore whether armed oncolytic virotherapy with the CXCR4 antagonist fused in-frame with the Fc portion of murine IgG2a delivered prior to immunization would improve the vaccine’s efficacy (Figure S4). In the pilot study, we analyzed the inhibition of tumor growth by the armed OVV-CXCR4-A-Fc virus or OVV-Fc used as a specificity control. Intravenous injections of 10^8^ PFU of OVV-Fc or OVV-CXCR4-A-Fc were initiated when the subcutaneous tumor volume was ~100 mm^3^. This titer of the OVV was used because it was found to be most efficacious in inhibiting tumor growth with no toxicity in adult tumor-bearing mice [43]. Untreated tumor-bearing mice served as controls. As shown in Figure 2C, the tumor burden after OVV-CXCR4-A-Fc treatment was significantly reduced compared with control (*p* < 0.001) and OVV-Fc-treated mice (*p* = 0.026), resulting in dormancy that extended for a period of over 1 week in the majority of treated mice (Figure 2D).

**Figure 3:**
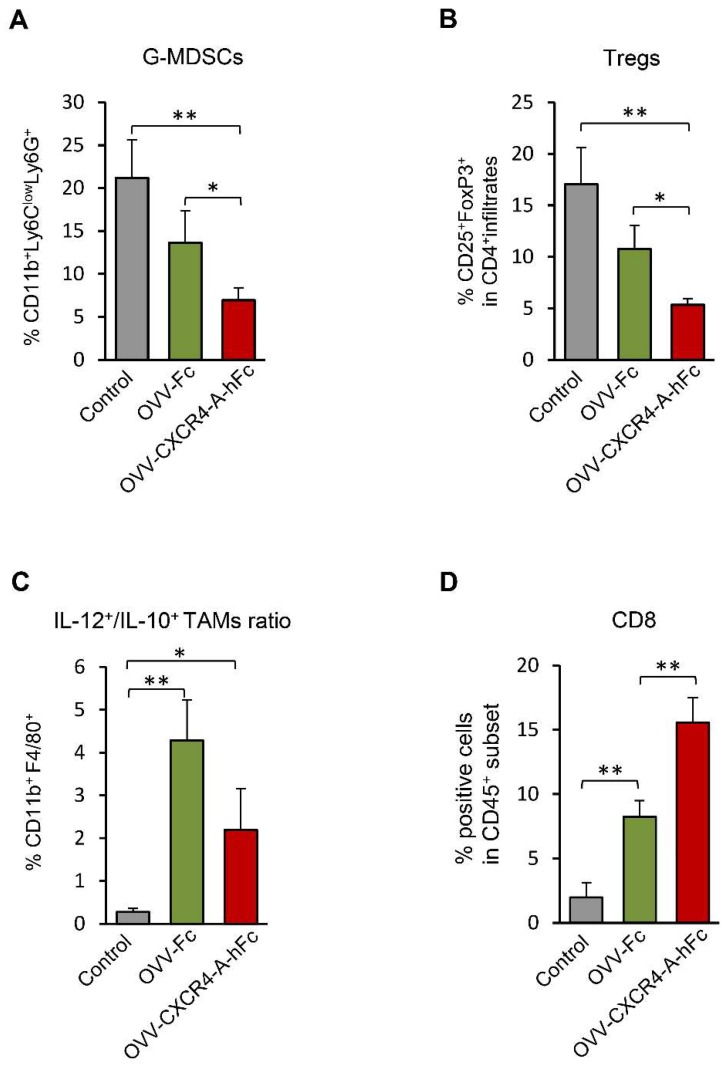
An armed OVV induces changes within the tumor microenvironment (TME) by targeting the CXCL12/CXCR4-signaling axis. The TME analysis performed on day 8 after completion of treatments. Frequencies of G-MDSCs (CD11b^+^Ly6C^low^Ly6G^+^) (**A**), and Tregs (CD4^+^CD25^+^Foxp3^+^) (**B**), were determined by flow cytometry staining performed on single-cell suspensions prepared from tumor tissues taken from control and treated mice (Materials and Methods section). Results are presented as mean ± SD of five mice per group. * *p* < 0.05, ** *p* < 0.01. The percentages of CD11b^+^F4/80^+^ monocytes/macrophages in tumor lysates of the same groups of mice as above were analyzed by flow cytometry. The expression of IL-10 and IL-12 in CD11b^+^F4/80^+^ cells was determined by intracellular staining. Results are presented as ratios of IL-12-producing versus IL-10-producing CD11b^+^F4/80^+^ tumor-associated macrophages (TAMs) (**C**). Data points represent mean ± SD. The percentage of CD8^+^ (**D**) tumor-infiltrating T lymphocytes (TILs) was determined by flow cytometry on single-cell suspensions prepared from tumor tissues taken from the same groups of mice. Background staining was assessed using isotype control antibodies. Results are presented as mean ± SD of five mice per group. * *p* < 0.05, ** *p* < 0.01.

Because the virally-delivered CXCR4 antagonist blocks the CXCL12/CXCR4 axis involved in tumor progression via enhanced local immunosuppression [15,16,42], we next investigated whether inhibition of NSX2 tumor after targeting the CXCL12/CXCR4-signaling axis through an oncolytic virus would also be related to changes within the TME (Figure 3). The analysis performed on day 8 after completion of treatments revealed that the inhibition of tumor growth in NXS2-bearing mice treated with OVV-CXCR-A-Fc was associated with reduction of intratumoral recruitment of granulocyte-like myeloid derived suppressor cells—G-MDSC (CD11^+^Ly6C^low^Ly6G^+^) compared to control and OVV-Fc treated mice (Figure 3A). Similarly, the number of regulatory T cells—Tregs (CD4^+^CD25^+^Foxp3^+^) in tumors treated with the armed virus was also reduced (Figure 3B) and these changes were associated with increased ratios of IL-12-producing versus IL-10-producing CD11b^+^F4/80^+^ inflammatory monocytes/macrophages (Figure 3C). The changes were associated with higher numbers of CD8^+^ tumor-infiltrating T lymphocytes (TILs) (Figure 3D), indicating that the reprogramming of the TME by the armed virus along with reduction of tumor load may improve the efficacy of DC vaccines.

### 3.4. OVV-CXCR4-A-Fc Contributes to Increased Efficacy of the NXS2 Tumor Lysate-Loaded Therapeutic DC Vaccines 

**Figure 4:**
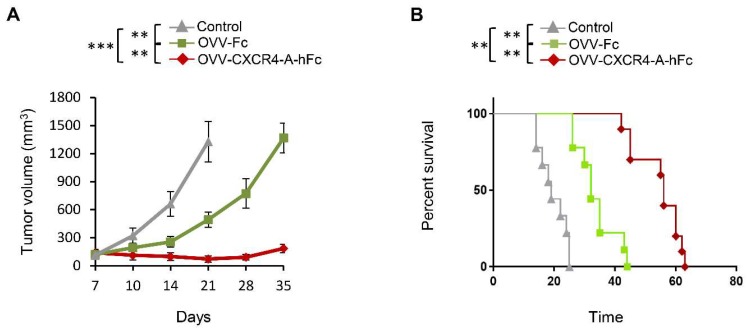
The efficacy of the NXS2 tumor lysate-loaded therapeutic DC vaccines is augmented by OVV-CXCR4-A-Fc treatment. A/J mice (n = 5/group) were challenged with 2 × 10^6^ NXS2 cells. The tumor cells were injected SC in the lateral flank. Oncolytic virotherapy with OVV-Fc and OVV-CXCR4-A-hFc delivered intravenously (10^8^ PFU/mouse) was initiated when the subcutaneous tumor volume was ~100 mm^3^. OVV-Fc was used as a specificity control. Control mice were treated with PBS. The Dox-treated NXS2 tumor cell lysates-loaded therapeutic DC vaccines were injected intradermally (2 × 10^6^ DCs/dose) on day 8 after the viral treatment when the cessation of viral replication occurred. (**A**) Tumor progression was monitored by measuring SC tumor growth with a microcaliper until control mice were euthanized due to extensive tumor burden. Results are presented as mean ± SD of five independent experiments. ** *p* < 0.01, *** *p* < 0.001. (**B**) Survival was defined as the point at which mice were killed because of extensive tumor burden. Kaplan–Meier survival plots were prepared, and significance was determined using the log-rank method. ** *p* < 0.01.

Although the single oncolytic virotherapy treatment with an armed OVV led to a significant reduction in tumor growth, the presence of residual tumors together with the significant changes in the TME prompted us to examine whether delivery of DCs pulsed with Dox-treated NXS2 tumor cell lysates would lead to improved disease-free survival (Figure S5). The DC vaccines were delivered to the tumor-bearing mice on day 8 after the oncolytic virotherapy when the cessation of viral replication occurred. Figure 4A shows that the DC vaccines delivered to tumor-bearing mice after treatment with OVV-Fc significantly reduced tumor growth compared to the therapeutic vaccines alone (*p* = 0.004). However, the highest efficacy of the combined treatment was achieved using the armed OVV-CXCR4-A-Fc virus prior to the vaccine delivery, suggesting that release of the CXCR4-A-Fc from virally-infected tumor cells into the tumor stroma significantly increased efficacy of the combined treatment compared to that achieved with the control virus (*p* = 0.002). This treatment resulted in dormancy that extended for a period of over 3 weeks in the majority of treated mice (Figure 4B). The results indicate the ability of the combined armed virus and DC vaccine treatment to promote the generation of protective antitumor immune responses.

## 4. Discussion

The SDF-1/CXCR4 pair is emerging as an increasingly important intermediary for the interaction between tumor cells and their environment, promoting both the metastatic disease as well as recruitment of immunosuppressive elements to the tumor [11,15,16,42]. Thus, the curative potential of therapies against NB hinges on eradicating the highly aggressive malignant cells in addition to countering the tumor immunosuppressive network [4]. We showed for the first time that targeted therapy of NB tumors with the CXCR4-A-Fc antagonist delivered by OVV not only reduced tumor load in the syngeneic tumor-bearing mice, but also reprogrammed the TME to effectively augment the efficacy of whole tumor cell lysate-loaded DC vaccines by producing a more permissive environment for induction of antitumor immunity. Importantly, this approach can also be used with other oncolytic viral vectors that are capable of inducing ICD in tumor cells to augment the release of tumor antigens available for cross-priming and increase the diversity of DAMPs to stimulate higher innate immune responses. Alternative approaches may also include expansion of intratumoral DCs following CXCR4 antagonist-armed oncolytic virotherapy treatment to increase phagocytic clearance of tumor cell debris after virally-mediated cytolysis.

Whole tumor lysates are especially important for inducing long-lasting antitumor immune responses by DC vaccines as they represent a promising source of T-cell epitopes and offer distinct advantages in tumor vaccine preparation [44]. For example, while whole protein antigens or recombinant viruses encoding the antigen of choice allow host major histocompatibility complex (MHC) molecules to select the appropriate peptide epitope for presentation on the cell surface, the spectrum of epitopes recognized by T-cells might be restricted because certain peptides are not presented by DCs due to missing processing at the level of the proteasome [45]. Ineffective cross-presentation of large protein antigens together with low transfection efficiency using cDNA or RNA are among several disadvantages to be considered. Furthermore, most of the identified tumor-associated peptides are human leukocyte antigen (HLA)-A2-restricted, implying that patients would be selected as eligible for DC-based therapy only according to their HLA-A2 status. Moreover, in view of the high rates of mutations in tumor cells and the loss of single or multiple epitopes, the restricted repertoire of immune responses peptides used for immunization might be insufficient for inhibiting tumor growth. On the other hand, whole tumor lysates offer a source of antigens that can elicit a tumor-specific response directed against multiple antigenic epitopes presented to both CD4^+^ and CD8^+^ T-cells for generation of long-term memory. Thus, although most of the current effort in cancer research concentrates on generating effective immune responses against model or well-defined antigens, vaccines targeting defined antigens have been clinically less successful than those based on whole tumor cells or their extracts [46].

The major caveat in using whole tumor lysates is that tumor cells are generally poorly immunogenic. In addition, the tumor cell lysates can be prepared in several ways and the methods for inducing immunogenic cell death that could impact phagocytosis of tumor cell debris and consequently the treatment efficacy remain to be elucidated. There are some approaches to improve the presentation of whole tumor lysate-peptides by MHC class I by induction of heat shock proteins that increase the presentation of exogenous peptides via class I molecules [47]. Additional parameters, including injection route, maturation state, and amount of antigen loaded onto administered DCs influence migration of these cells to the lymph nodes, and thus their ability to trigger an effective cytotoxic immune response [48,49]. Consistent with previous studies, we showed that expression of CRT on Dox-treated tumor cells, which subsequently succumbed to ICD, was associated with increased phagocytosis of tumor cell debris by DCs. The cytosolic localization of antigens following 24-h co-culture with whole Dox-treated tumor lysates confirmed that efficient antigen transfer resulting in the cytoplasmic localization of tumor-associated antigens was accomplished in DCs and required to achieve meaningful antitumor efficacy in a prophylactic setting. However, these potent whole tumor lysate-loaded DC vaccines were rather ineffective in inhibiting tumor growth in NXS2-challenged mice. The latter effect might be attributed to tumor burden as well as upregulation of immunosuppressive mechanisms in the TME in response to immune attack, termed adaptive immune resistance [11,12], stressing the need for identifying approaches to bolster antitumor immunity and simultaneously limit immune suppression in the TME. We presented here that pretreatment of NXS2 tumor-bearing mice with OVV mediated direct oncolysis, leading to significant reduction of tumor load. This, together with the ability of the virus to elicit innate immunity through the TLR2/MyD88-dependent pathway and TLR independent production of IFN-β [25,26] could produce a more permissive environment for antitumor immunity. Although reduction of tumor load is an important factor in determining the efficacy of therapeutic antitumor vaccines, a still-unresolved issue surrounding tumor growth involves the role that the immune system plays in resisting or eradicating the formation and progression of tumors [50]. During this process, cancer cells may paralyze infiltrating cytotoxic T lymphocytes (CTLs) by secreting immunosuppressive factors [51] or by more subtle mechanisms that operate through the recruitment of immunosuppressive elements, including MDSCs and Tregs. In this study, we demonstrated that targeting the CXCL12/CXCR4 migratory axis with the virally expressed CXCR4 antagonist inhibited intratumoral accumulation of immunosuppressive MDSCs and Tregs, and increased IL-12 to IL-10-producing TAMs. These changes within the TME had a significant impact on the antitumor efficacy of the DC vaccines. The presented mechanism of inhibition of immunosuppressive pathways promoting tumor growth prior to vaccine delivery is likely applicable to different cancer types and can potentially unravel novel therapeutic avenues that could significantly enhance the efficacy of therapeutic DC vaccination. 

## 5. Conclusions

In conclusion, our results indicate that the use of CXCR4 antagonist-armed oncolytic virotherapy with vaccinia virus represents a suitable approach to activate innate and adaptive immune responses at the tumor site, allowing the achievement of therapeutic effects in a highly tumorigenic neuroblastoma model.

## Supplementary Materials

The following are available online at http://www.mdpi.com/1999-4915/10/9/455/s1. Figure S1. Testing the immunogenicity of DC vaccines—prophylactic setting. The tumor lysates-pulsed murine DCs (2 × 10^6^ DCs/dose) or untreated DCs (2 × 10^6^ DCs/dose) were injected intradermally into A/J mice (*n* = 5/group). Next, each mouse was injected SC in the lateral flank with 2 × 10^6^ NXS2 cells on day 8 after vaccination (day 0). Unvaccinated mice (*n* = 5) served as a control group. Tumor growth was monitored by measuring SC tumors once to thrice a week with a microcaliper until control mice were euthanized due to extensive tumor burden. * *p* < 0.05, ** *p* < 0.01, *** *p* < 0.001. Figure S2. Effect of DC vaccines on tumor growth of NXS2 tumor-challenged SCID mice. BM-derived DCs were loaded with Dox-treated NXS2 tumor cell lysates or untreated NXS2 tumor cells and injected intradermally (2 × 10^6^ DCs/dose) into SCID mice. Mice were challenged SC in the lateral flank with 2 × 10^6^ NXS2 cells on day 8 after vaccination. Control mice were unvaccinated. Tumor growth was monitored by measuring SC tumor growth with a microcaliper until control mice were euthanized due to extensive tumor burden. Results are presented as mean ± SD of three independent experiments. Figure S3. Testing the immunogenicity of DC vaccines—therapeutic setting. A/J mice (*n* = 15) were first injected SC with 2 × 10^6^ NXS2 cells in the lateral flank and 7 days later DC vaccines (DCs loaded with NXS2 lysates preapared from untreated or Dox-treated tumor cells) were injected intradermally into the opposite site (*n* = 5/group). Unvaccinated A/J mice served as controls (*n* = 5). Tumor growth was monitored by measuring SC tumors once to thrice a week with a microcaliper until control mice were euthanized due to extensive tumor burden. Figure S4. Treatment of established tumors with oncolytic viruses. A/J mice (*n* = 15) were injected SC with 2 × 10^6^ NXS2 cells and treated with OVV-CXCR4-A-Fc or OVV-Fc (10^8^ PFU delivered IV; *n* = 5 mice per group) once the tumor volumes reached ∼100 mm^3^ (on day 7). Control mice (*n* = 5) received PBS or UV-inactivated virus. Tumor progression was monitored by measuring SC tumor growth with a microcaliper until control mice were euthanized due to extensive tumor burden. * *p* < 0.05, ** *p* < 0.01, *** *p* < 0.001. **Figure S5**. The enhancement of oncolytic viruses treatment of established tumors with DC vaccines. NXS2 tumor-bearing mice were treated with OVV-CXCR4-A-Fc or OVV-Fc (10^8^ PFU delivered IV) once the tumor volumes reached ∼100 mm^3^ (on day 7). Control group was injected with PBS or UV-inactivated virus (*n* = 5 mice/group). The DC vaccines were delivered after the cessation of viral replication (on day 14). Tumor growth was monitored by measuring SC tumors once to thrice a week with a microcaliper until control mice were euthanized due to extensive tumor burden. ** *p* < 0.01, *** *p* < 0.001.
