# Supplementary material for: Modulation of the Tumor Microenvironment by CXCR4 Antagonist-Armed Viral Oncotherapy Enhances the Antitumor Efficacy of Dendritic Cell Vaccines against Neuroblastoma in Syngeneic Mice
Source: Viruses. 2018 Aug 26;10(9):455. doi: 10.3390/v10090455 (PMC6165252; doi:10.3390/v10090455)
Supplement: Supplementary file 1 [file viruses-10-00455-s001.pdf]

Supplementary Materials.

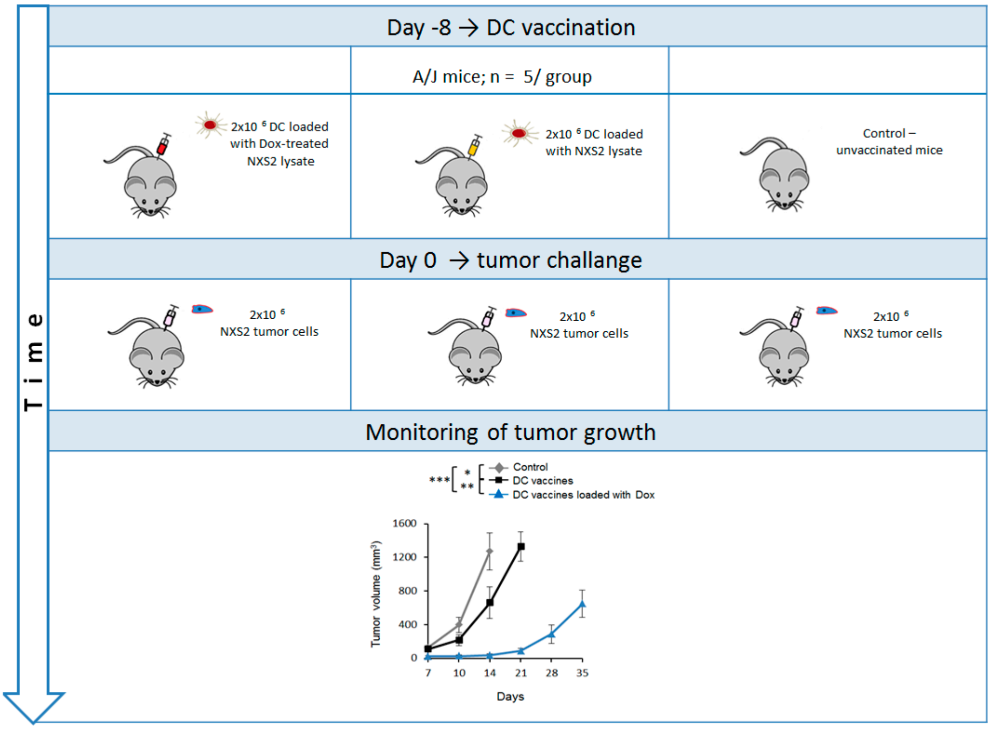

Figure S1. Testing the immunogenicity of DC vaccines – prophylactic setting. The tumor lysates-pulsed murine DCs (2 x 10^6 DCs/dose) or untreated DCs (2 x 10^6 DCs/dose) were injected intradermally into A/J mice (n = 5/group). Next, each mouse was injected subcutaneously (SC) in the lateral flank with 2 x 10^6 NXS2 cells on day 8 after vaccination (day 0). Unvaccinated mice (n = 5) served as a contol group. Tumor growth was monitored by measuring SC tumors once to thrice a week with a microcaliper until control mice were euthanized due to extensive tumor burden. $*p < 0.05$, $**p < 0.01$, $***p < 0.001$.

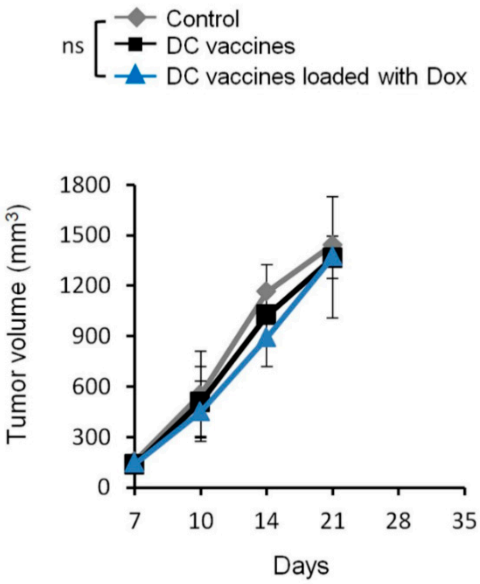

Figure S2. Effect of DC vaccines on tumor growth of NXS2 tumor-challenged SCID mice. BM-derived DCs were loaded with Dox-treated NXS2 tumor cell lysates or untreated NXS2 tumor cells and injected intradermally (2 x 10^6 DCs/dose) into SCID mice. Mice were challenged SC in the lateral flank

with 2 x 10⁶ NXS2 cells on day 8 after vaccination. Control mice were unvaccinated. Tumor growth was monitored by measuring SC tumor growth with a microcaliper until control mice were euthanized due to extensive tumor burden. Results are presented as mean ± SD of three independent experiments.

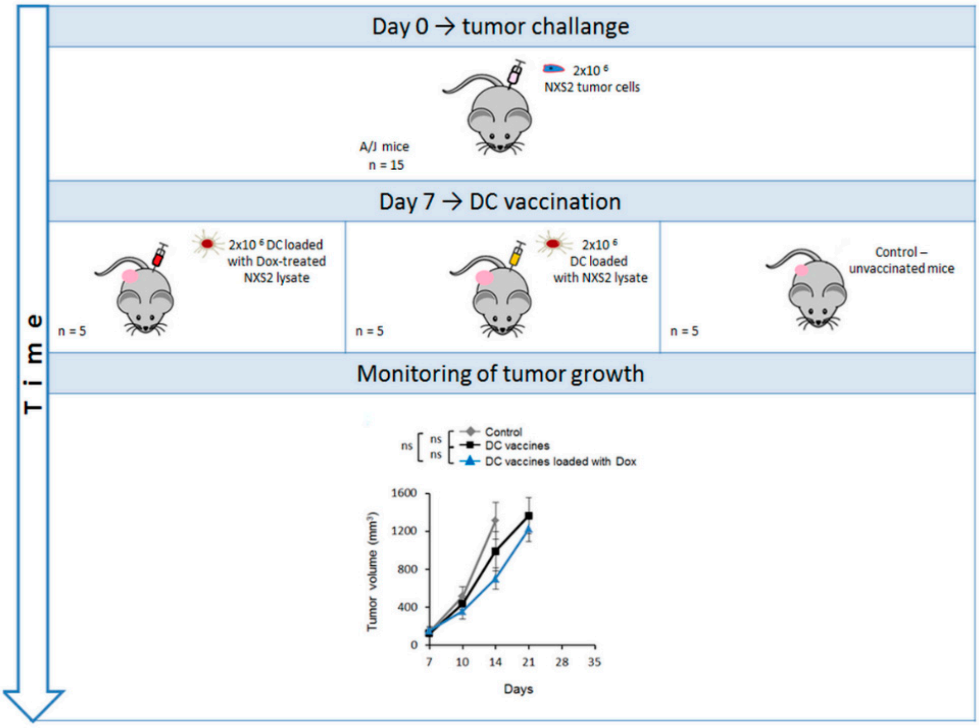

Figure S3. Testing the immunogenicity of DC vaccines—therapeutic setting. A/J mice (*n*= 15) were first injected SC with 2 x 10⁶ NXS2 cells in the lateral flank and 7 days later DC vaccines (DCs loaded with NXS2 lysates preapared from untreated or Dox-treated tumor cells) were injected intradermally into the opposite site (*n* = 5/group). Unvaccinated A/J mice served as controls (*n* = 5). Tumor growth was monitored by measuring SC tumors once to thrice a week with a microcaliper until control mice were euthanized due to extensive tumor burden.

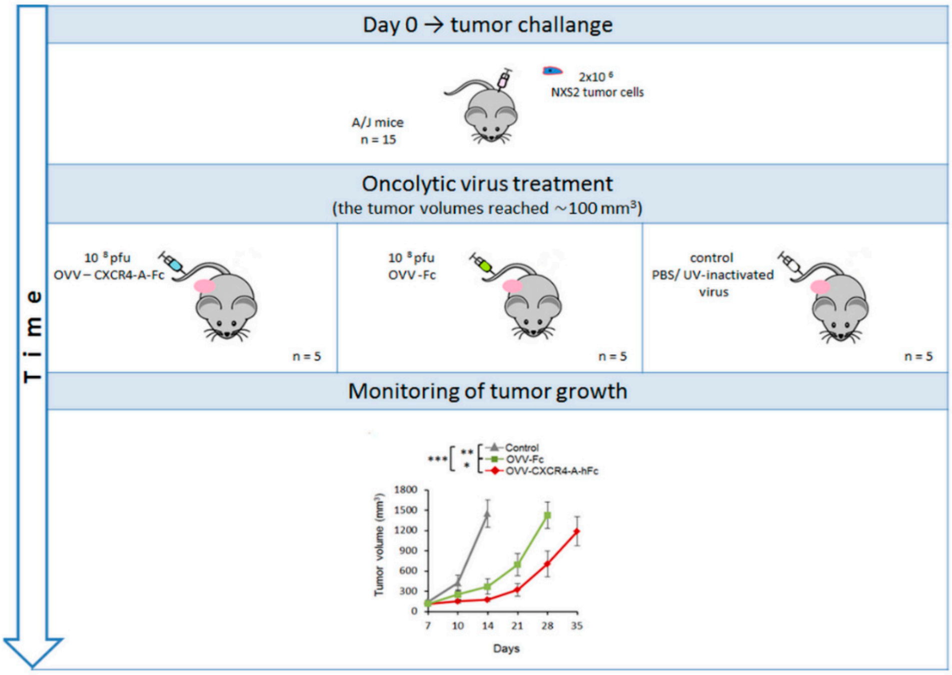

Figure S2. Treatment of established tumors with oncolytic viruses. A/J mice (n = 15) were injected SC with 2 x 10^6 NXS2 cells and treated with OVV-CXCR4-A-Fc or OVV-Fc (10^8 PFU delivered intravenously, IV; n = 5 mice per group) once the tumor volumes reached ~100 mm^3 (on day 7). Control mice (n=5) received PBS or UV-inactivated virus. Tumor progression was monitored by measuring SC tumor growth with a microcaliper until control mice were euthanized due to extensive tumor burden. *$p < 0.05$, **$p < 0.01$, ***$p < 0.001$.

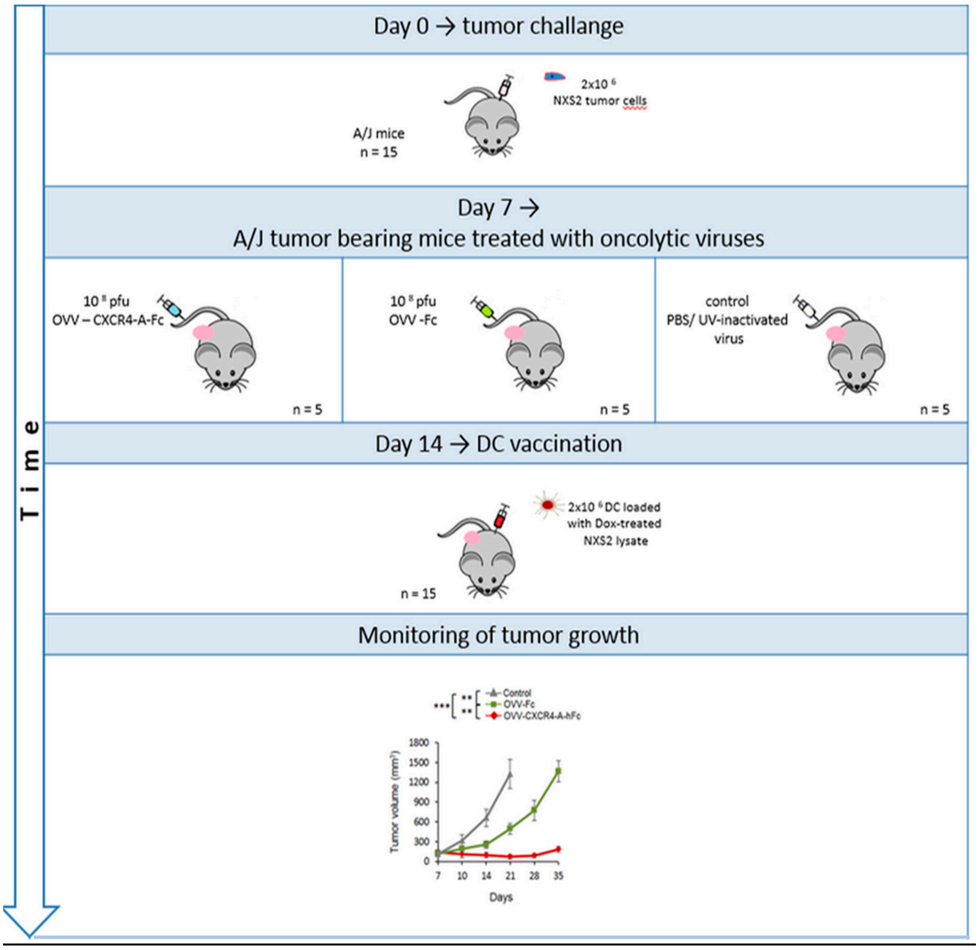

Figure S3. The enhancement of oncolytic viruses treatment of established tumors with DC vaccines. NXS2 tumor bearing mice were treated with OVV-CXCR4-A-Fc or OVV-Fc (10^8 PFU delivered IV) once the tumor volumes reached ~100 mm^3 (on day 7). Control group was injected with PBS or UV inactivated virus (n = 5 mice/group). The DC vaccines were delivered after the cessation of viral replication (on day 14). Tumor growth was monitored by measuring SC tumors once to thrice a week with a microcaliper until control mice were euthanized due to extensive tumor burden. **$p < 0.01$, ***$p < 0.001$.
